# *Echinococcus granulosus* cyst fluid inhibits inflammatory responses through inducing histone demethylase KDM5B in macrophages

**DOI:** 10.1186/s13071-023-05948-1

**Published:** 2023-09-09

**Authors:** Xiaopeng Wang, Ruolin Lin, Chunxue Fu, Chun Yang, Dan Dong, Xiangwei Wu, Xueling Chen, Lianghai Wang, Jun Hou

**Affiliations:** 1 NHC Key Laboratory of Prevention and Treatment of Central Asia High Incidence Diseases, The First Affiliated Hospital, Shihezi University School of Medicine, Shihezi, Xinjiang China; 2 Key Laboratory of Xinjiang Endemic and Ethnic Diseases, Shihezi University School of Medicine, Shihezi, Xinjiang China

**Keywords:** Cystic echinococcosis, KDM5B, Macrophages, Histone modification

## Abstract

**Background:**

*Echinococcus granulosus* cyst fluid (EgCF) weakens macrophage inflammatory responses, thereby enabling the parasite to evade the immune system. However, the role of histone modification in this process remains to be explored.

**Methods:**

The levels of IL-6, TNF-α, IL-10, H3K4me3, and KDM5B were detected using quantitative real-time PCR, ELISA, and Western blotting. The enrichment of H3K4me3 and KDM5B at the promoter of inflammatory factors was detected by chromatin immunoprecipitation.

**Results:**

Based on EgCF-stimulated macrophage models, we found that EgCF significantly inhibited mRNA expression and protein secretion of IL-6 and TNF-α and upregulated mRNA expression of IL-10 under the influence of TLR4. EgCF lowered the level of H3K4me3 and promoted the transcription and protein stability of histone demethylase KDM5B. Chromatin immunoprecipitation analysis revealed that EgCF suppressed the enrichment of H3K4me3 modification at the promoters of TNF-α and IL-6 and downregulated their expression in macrophages. Additionally, the inhibition of KDM5B activity by CPI-455 weakened the anti-inflammatory effect of EgCF.

**Conclusions:**

Our findings demonstrate a novel mechanism through which EgCF promotes KDM5B expression and inhibits the enrichment of H3K4me3 at the promoters of inflammatory cytokines to suppress the inflammatory response.

**Graphical Abstract:**

**Figure:**
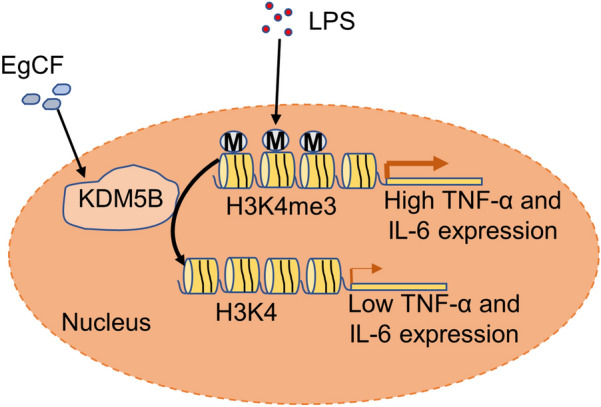

**Supplementary Information:**

The online version contains supplementary material available at 10.1186/s13071-023-05948-1.

## Introduction

*Echinococcosis* refers to two serious zoonoses, which are mainly defined as cystic echinococcosis (CE) and alveolar echinococcosis caused by *Echinococcus granulosus* and *E. multilocularis* in humans, respectively [1, 2]. Generally, the CE cyst includes the parasite larva which establishes in the intermediate host (mainly domestic ungulates); the definitive host (carnivore) contains the adult worm [3]. Humans may become an intermediate host of *Echinococcus granulosus* due to inadvertent ingestion of parasite eggs present in contaminated water or food. Established fluid-filled CE cysts are primarily located in the liver and lung [1, 4]. Surgery is the primary option for treating echinococcosis, but surgery can also cause secondary cysts [1, 5]. In addition, early diagnosis of echinococcosis remains difficult, and drugs may cause adverse events [1]. Therefore, it is imperative to explore the pathogenic mechanisms of echinococcosis and develop novel anti-parasitic treatments.

Long-term survival of *Echinococcus granulosus* in the host suggests that the parasite has evolved certain mechanisms to avoid immune response from the host [4]. Mechanisms have not been understood yet but some larval components are candidates as they showed immunomodulatory effects on innate immune cells [6]. Notably, *E. granulosus* cyst fluid (EgCF) is a complex mixture of components that could modulate immune responses [7, 8]. EgCF contains many secreted proteins helpful to parasite survival [9]. For instance, glycomolecules in the EgCF can interfere with dendritic cell maturation and activation mediated by the TLR4 signaling pathway [10–12].

Macrophages are critical mediators of pathogen-driven escape [13]. Depending on the microenvironment, macrophages can acquire distinct functional phenotypes including classically activated macrophages (M1 macrophages) and alternatively activated macrophages (M2 macrophages) [14]. Under the influence of LPS, macrophages can be polarized to the M1 phenotype and secrete high levels of pro-inflammatory cytokines such as TNF-α and IL-6 [14]. Recent evidence suggests that the function of macrophages can be regulated by histone modifications [15], which are dynamically regulated by histone-modifying enzymes [16]. KDM5B as a histone demethylase belongs to the KDM5 family [17]. It is a JmjC-containing demethylase that selectively targets H3K4me3 [15], a well-known marker of active transcription [18]. KDM5B can inhibit transcription factor NF-κB binding to IL-6 and IL-23a promoters through demethylating H3K4me3 in bone marrow-derived macrophages (BMDMs) [19]. However, the function of KDM5B in macrophages during cystic echinococcosis is poorly understood.

This study investigated the role of histone modification in suppressing macrophage inflammatory responses resulting from EgCF. The current results showed that EgCF promoted the transcription and protein stability of KDM5B, which inhibited the enrichment of H3K4me3 at the promoters of inflammatory factors and suppressed the inflammatory response.

## Materials and methods

### Cell culture

The mouse macrophage cell line RAW 264.7 and the human monocyte cell line THP-1 were purchased from the Cell Bank of Shanghai Institutes for Biological Sciences, Chinese Academy of Sciences and Wuhan Procell Life Science and Technology Co., Ltd., respectively. RAW 264.7 cells were maintained in DMEM (Gibco) containing 10% heat-inactivated fetal bovine serum (FBS, Gibco) and 1% penicillin-streptomycin (Gibco) in a humidified atmosphere with 5% CO_2_ at 37 °C following the provider’s instructions. THP-1 cells were cultured in RPMI 1640 (Gibco) supplemented with 10% FBS, 1% penicillin-streptomycin, and 0.05 mM β-mercaptoethanol (Gibco). THP-1 cells were differentiated into macrophages by treating the cells with 100 nM phorbol 12-myristate 13-acetate (PMA) for 24 h (h) [20–23].

Mouse peritoneal macrophages were isolated from C57BL/6 female mice. Briefly, the mice were killed and intraperitoneally injected with DMEM using a 5-ml syringe. The abdomen was gently massaged for 5 min without removing the needle. Subsequently, the fluid was slowly withdrawn from the abdominal cavity via the syringe. The collected peritoneal fluid was centrifuged at 300 g for 5 min. Next, the precipitated cells were washed and seeded into six-well plates at a density of 1 × 10^6^ cells per well in 2 ml medium (DMEM supplemented with 1% l-glutamine, 10% FBS, and 1% penicillin-streptomycin). Non-adherent cells were removed after 2-h culture and fed with 2 ml of fresh medium. The adherent cells were treated with LPS and EgCF the following day, as previously reported [12, 24].

### Preparation of *Echinococcus granulosus* (sensu lato) cyst fluid

Sheep liver CE cysts were obtained from a slaughterhouse in Shihezi, Xinjiang, China. Briefly, the liver and operating instruments were sterilized using 75% alcohol. The fluid was withdrawn from the cysts using a sterile 50-ml syringe and fully mixed. After standing, the clear and transparent cyst fluid in the upper layer was centrifuged at 1000 g for 10 min and filtered through a 0.22-μM filter. The EgCF employed was a pool generated from different cysts and stored in a − 80 °C freezer after protein concentration had been estimated by NanoDrop (4 mg/ml).

### Quantitative real-time PCR

RNA was extracted using a total RNA kit (Omega) and reverse-transcribed using HiFiScript cDNA Synthesis Kit (Cwbio) following the manufacturer’s instructions. Quantitative real-time PCR (qRT-PCR) was conducted utilizing the UltraSYBR One-Step RT-qPCR Kit (Cwbio) with specific primers in a CFX96 Touch detection system (Bio-Rad). Relative expression levels of genes were normalized to β-actin by the 2^−ΔΔCt^ method. Primer sequences are listed in Table 1.

**Table 1:** Primer sequences for qRT-PCR and ChIP assay

Symbol	Forward	Reverse
Mouse TNF-α	TGATCCGCGACGTGGAA	ACCGCCTGGAGTTCTGGAA
Mouse IL-6	GAGGATACCACTCCCAACAGACC	AAGTGCATCATCGTTGTTCATACA
Mouse KDM5B	ATCGCTTGCTGCACCGTTAT	CGCTCATCATCTGGCAACAG
Mouse KDM6B	ATAGGGCCCTTCGCTATGGA	CGTTTGTGCTCAAGGTGCAG
Mouse KDM7A	TGCAGCTCTACACGGCTCT	CCAGCTTGAACAGGTTTGGAG
Mouse IL-10	ACCTGGTAGAAGTGATGCC	CACCTTGGTCTTGGAGCT
Mouse β-actin	GGCTATGCTCTCCCTCACG	GAGCAACATAGCACAGCTTCTCTTT
Human IL-6	GATGGATGCTTCCAATCTGGAT	AGTTCTCCATAGAGAACAACATA
HumanKDM5B	AGTGGGCTCACATATCAGAGG	CAAACACCTTAGGCTGTCTCC
Human TNF-α	TTCCCCAGGGACCTCTCTCTAATC	GAGGGTTTGCTACAACATGGGCTAC
Human IL-10	GACTTTAAGGGTTACCTGGGTTG	TCACATGCGCCTTGATGTCTG
Human β-actin	GAGAAAATCTGGCACCACACC	GGATAGCACAGCCTGGATAGCAA
TNF-α promoter	CAGCCACTGCTTGGCTAGAC	CGGATCCCATGGACCAACTG
IL-6 promoter	AGGAGTGTGAGGCAGAGAGC	GTCTCCTCTCCGGACTTGTG

### ELISA

RAW 264.7 cells and differentiated THP-1 cells were treated with LPS (1 μg/ml) in the presence or absence of EgCF (50% v/v) for 24 h. The cell culture medium was collected and centrifuged at 1000 g for 10 min. The TNF-α and IL-6 concentrations in the harvested supernatants were measured using ELISA kits (Multi Sciences).

### Western blotting

Cells were washed and lysed in the RIPA lysis buffer. Protein lysates were electrophoretically separated on sodium dodecyl sulfate-polyacrylamide gel electrophoresis (SDS-PAGE) and then transferred to PVDF membranes. Five percent bovine serum albumin dissolved in Tris-buffered saline containing 0.1% Tween-20 (TBST) was used to block the membranes for 2 h. Next, incubation was performed at 4 °C overnight with primary antibodies specific to H3K4me3 (CST #9751), H3K9me3 (CST #13969), H3K36me3 (CST #4909), Histone H3 (BOSTER A12477-2), KDM5B (CST #15327), and β-actin (ZSGB-BIO #TA-09). After washing three times with TBST for 10 min, the membranes were further incubated with peroxidase-labeled secondary antibodies for 1 h at room temperature. The immune complexes were visualized with the ECL substrate (Biosharp BL520A).

### Chromatin immunoprecipitation

The chromatin immunoprecipitation (ChIP) assay was performed using the SimpleChIP Plus Enzymatic Chromatin IP Kit (Agarose Beads; CST #9002) according to the manufacturer’s instructions. Cells were cross-linked with a final concentration of 1% formaldehyde for 10 min, and the reaction was terminated by adding glycine at room temperature. The cells were washed with PBS and incubated in lysis buffer on ice for 10 min and disrupted using an ultrasonic cell disruptor. After centrifugation, chromatin was prepared by incubating with specific primary Ab and anti-IgG overnight at 4 °C and precipitated using ChIP-Grade Protein G Agarose Beads. Following the reversal of the protein-DNA crosslinking, DNA was purified using DNA Purification Buffers and Spin Columns (CST #14209). The enrichment of specific DNA sequences following immunoprecipitation was determined by semi-quantitative PCR (semi-qPCR) and qRT-PCR with primers listed in Table 1. Data were normalized to input DNA, and IgG was used as a negative control.

### Statistical analysis

All the experiments were independently repeated three times. Data were represented as mean + standard error of the mean (SEM). Statistical analysis was performed using one-way ANOVA for comparing differences between groups in GraphPad Prism (version 8). * *P* < 0.05, ** P < 0.01, and *** *P* < 0.001 indicate a statistically significant difference.

## Results

### EgCF inhibited inflammatory response mediated by TLR4 signaling pathway in macrophages

**Fig. 1:**
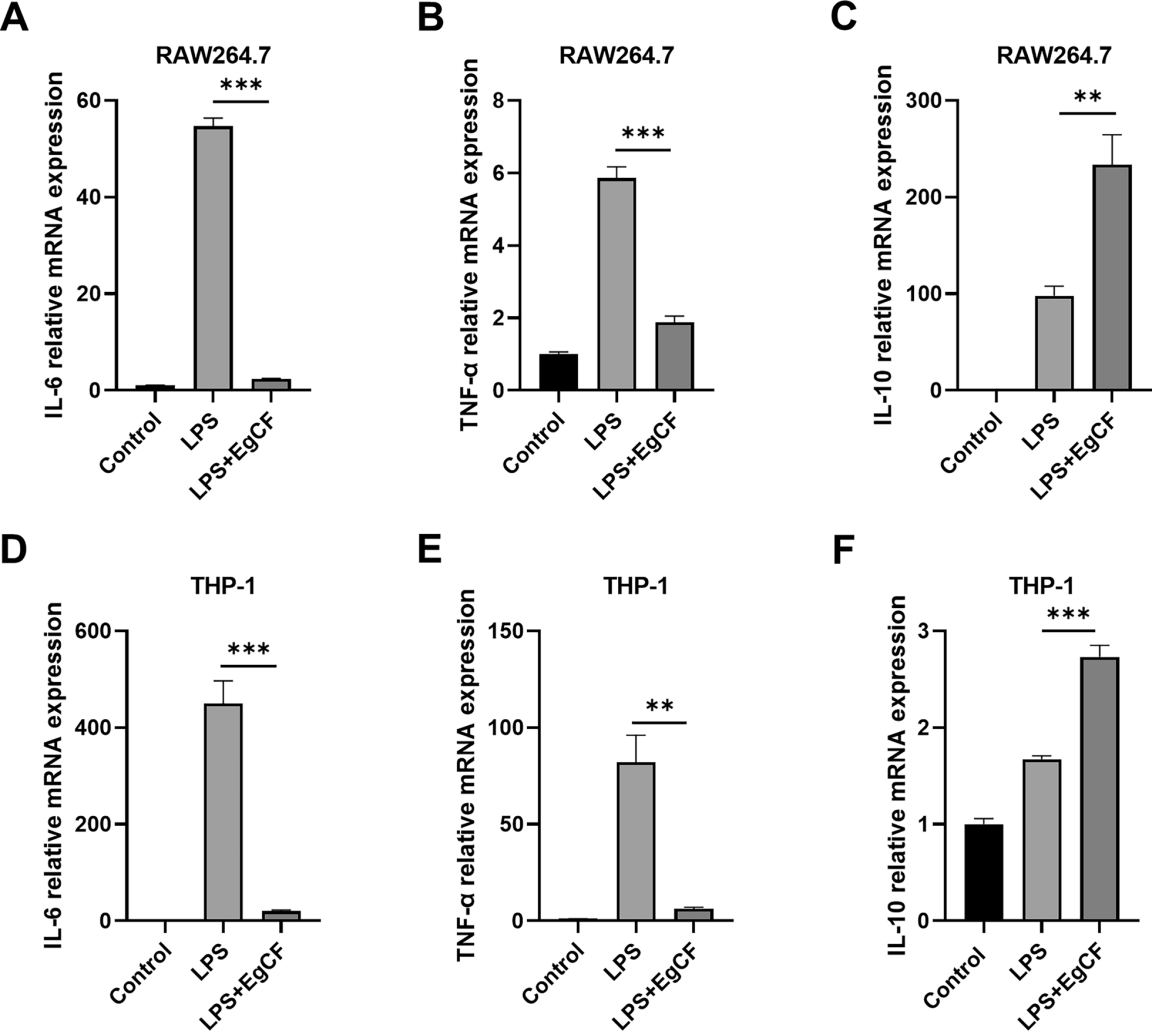
EgCF suppressed the mRNA expression of inflammatory factors in macrophages. RAW 264.7 cells were treated with EgCF (50% v/v) and LPS (1 μg/ml) for 6 h. The mRNA expression levels of IL-6 (**A**), TNF-α (**B**), and IL-10 (**C**) were detected by qRT-PCR. THP-1 cells were treated with EgCF (50% v/v) and LPS (1 μg/ml) for 6 h. The mRNA expression levels of IL-6 (**D**), TNF-α (**E**), and IL-10 (**F**) were detected by qRT-PCR. Data are presented as the mean + SEM of three independent experiments and compared using the one-way ANOVA and Tukey test. Asterisks indicate a significant difference at **P < 0.01 and ***P < 0.001

**Fig. 2:**
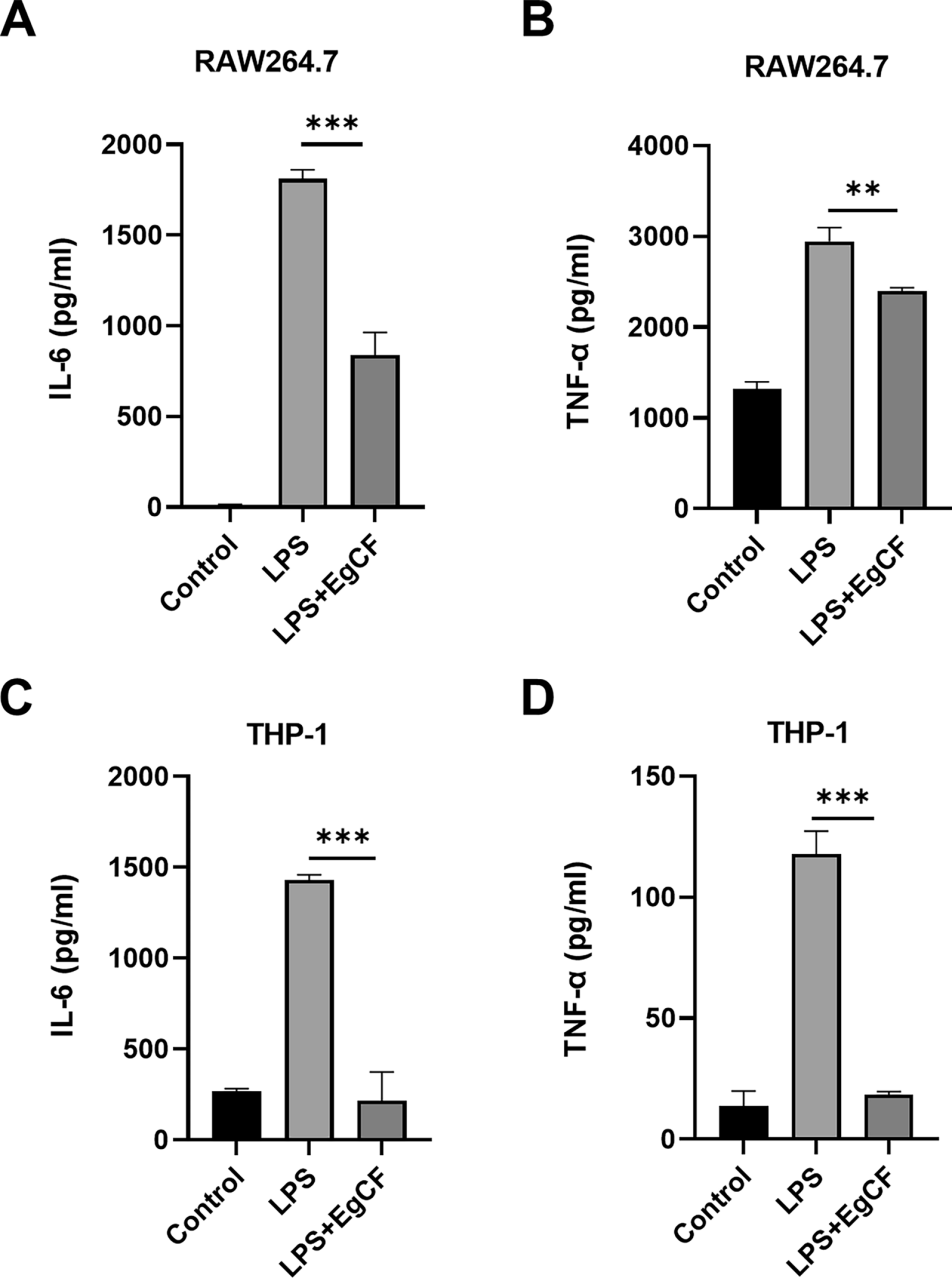
EgCF inhibited the secretion of inflammatory factors in macrophages. RAW 264.7 cells were treated with EgCF (50% v/v) and LPS (1 μg/ml) for 24 h. Secretion of IL-6 (**A**) and TNF-α (**B**) in cell culture supernatants was measured by ELISA. THP-1 cells were treated with EgCF (50% v/v) and LPS (1 μg/ml) for 24 h. Secretion of IL-6 (**C**) and TNF-α (**D**) in cell culture supernatants was measured by ELISA. Data are presented as the mean + SEM of three independent experiments and compared using the one-way ANOVA and Tukey test. Asterisks indicate a significant difference at **P < 0.01 and ***P < 0.001

We first checked cell viability after treatment for all cell sources to ensure effects were not due to cell death (Additional file 1: Fig. S1). Subsequently, we detected the mRNA expression level of IL-6, TNF-α, and IL-10 in mouse RAW 264.7 cells stimulated with LPS and EgCF for 6 h. qRT-PCR analysis showed that simultaneous stimulation of EgCF and LPS significantly inhibited the mRNA expression of IL-6 and TNF-α compared with using LPS stimulation alone (Fig. 1A and B). Contrarily, co-stimulation of LPS and EgCF upregulated the mRNA expression of IL-10 (Fig. 1C). Similarly, co-stimulation of EgCF and LPS in human THP-1 cells also markedly suppressed the mRNA expression of IL-6 and TNF-α and promoted the mRNA expression of IL-10 (Fig. 1D–F). Then, we detected IL-6 and TNF-α protein secretion in supernatants of RAW 264.7 cells and THP-1 cells stimulated with EgCF and LPS for 24 h. ELISA analysis demonstrated that compared with using LPS stimulation alone, the protein levels of IL-6 and TNF-α in cell supernatants were reduced after simultaneous stimulation of EgCF and LPS (Fig. 2A–D). These results indicated that EgCF inhibited macrophage inflammatory responses mediated by the TLR4 signaling pathway.

### EgCF suppressed H3K4me3 modification

**Fig. 3:**
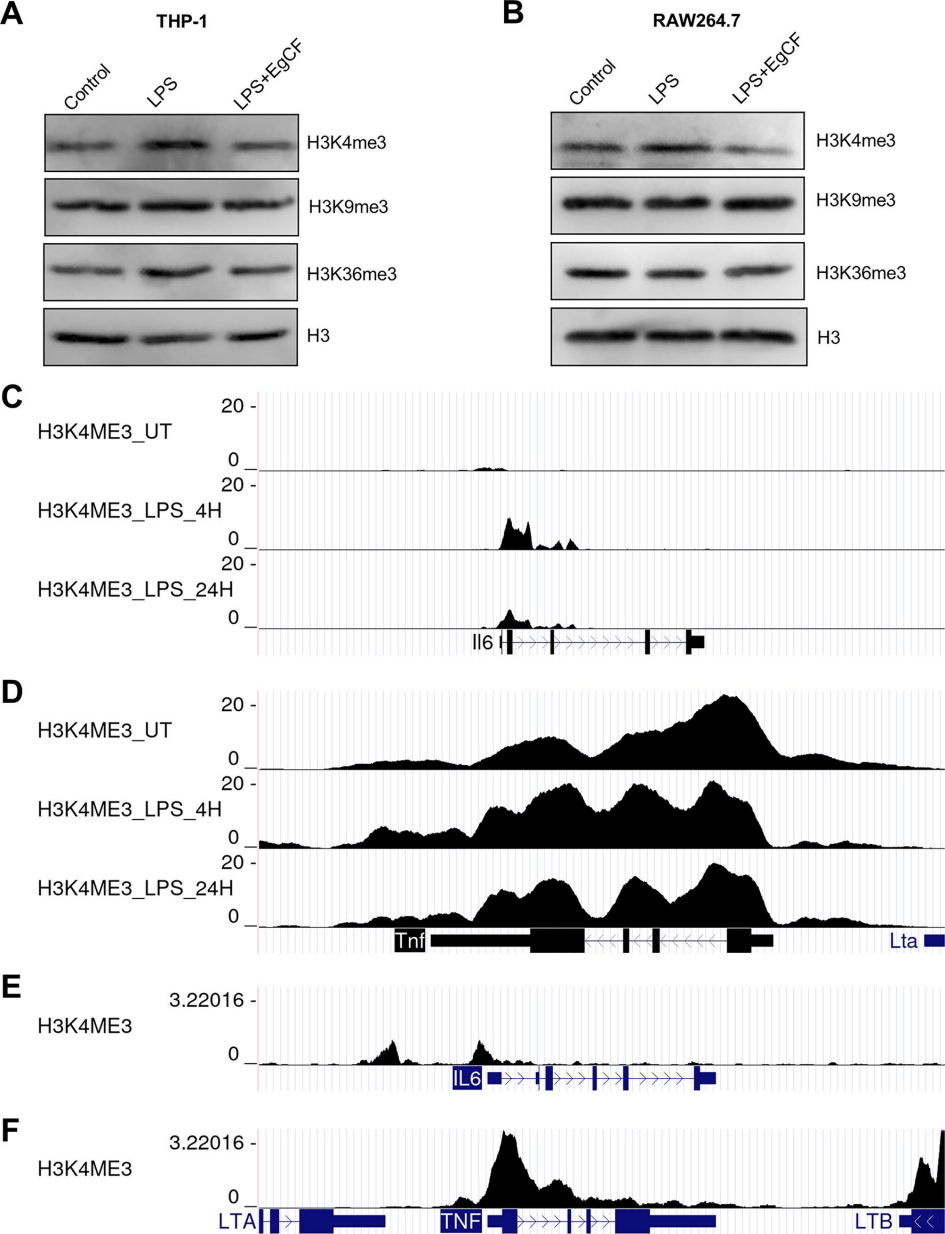
EgCF reduced H3K4me3 modification. THP-1 and RAW 264.7 cells were treated with EgCF (50% v/v) and LPS (1 μg/ml) for 6 h. Levels of H3K4me3, H3K9me3, and H3K36me3 were measured by Western blotting (**A**, **B**). Enrichment of H3K4me3 at IL-6 and TNF-α promoters in mouse bone marrow-derived macrophages (GSM2419196, GSM2419204, GSM2419211) (**C**, **D**) and human THP-1 cells (GSM2108047) (**E**, **F**)

Methylation of H3K4 and H3K36 typically suggests active transcription, whereas H3K9 methylation is associated with a silent chromatin state [25, 26]. THP-1 and RAW 264.7 cells were stimulated with EgCF and LPS. After 6 h of stimulation, the global levels of H3K4me3, H3K9me3, and H3K36me3 were detected by Western blotting. Our results indicated that the co-stimulation of LPS and EgCF inhibited the global levels of H3K4me3 compared with the stimulation of LPS alone, but there was no obvious difference in H3K9me3 or H3K36me3 level (Fig. 3A and  B). We also found a high enrichment of H3K4me3 modification at the promoter of IL-6 and TNF-α in human THP-1 cells and mouse BMDMs based on public chromatin immunoprecipitation and sequencing (ChIP-seq) data (Fig. 3C–F). These results indicated that EgCF interfered with H3K4me3 modification.

### EgCF promoted transcription and protein stability of KDM5B

**Fig. 4:**
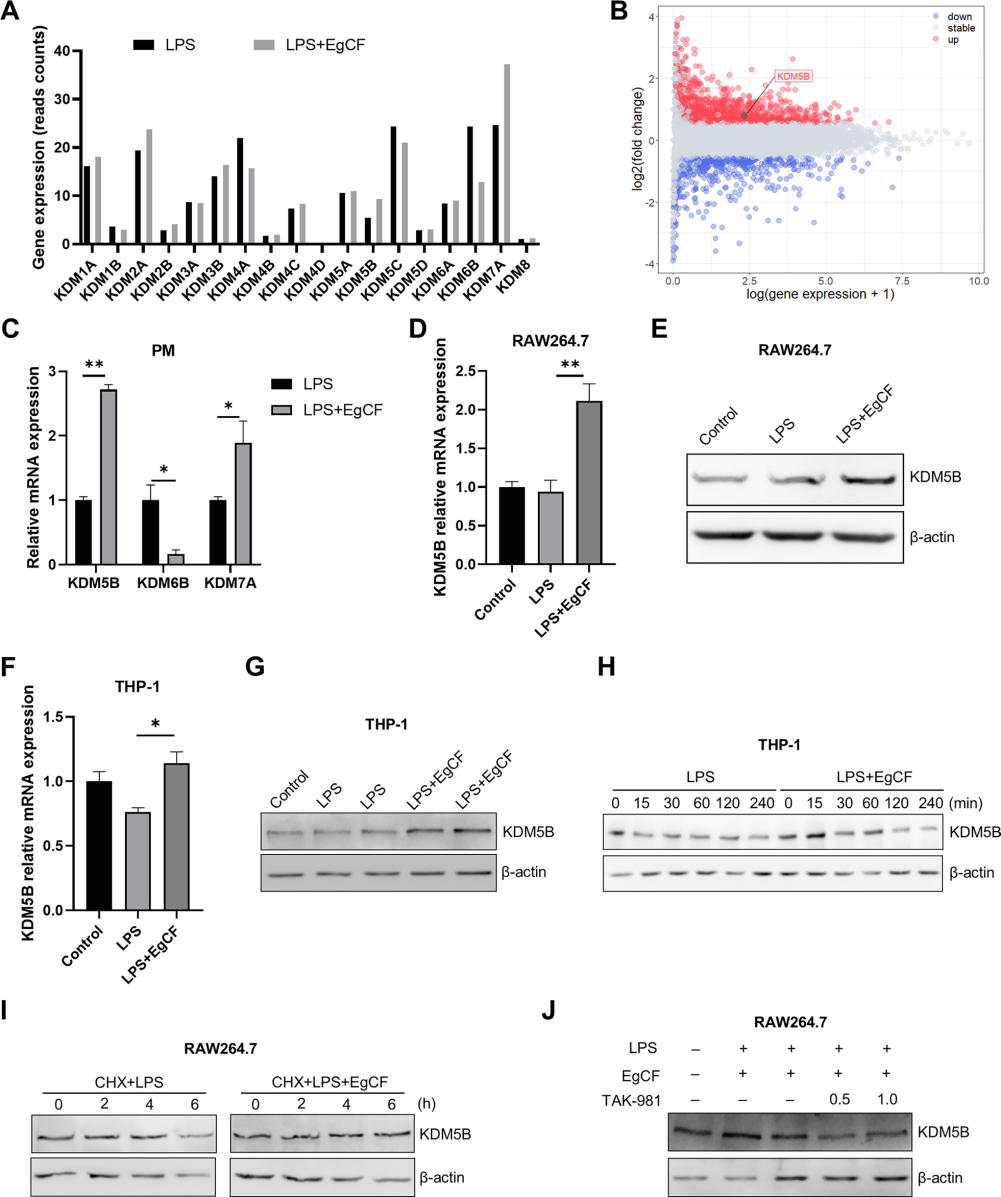
EgCF promoted transcription and protein stability of KDM5B. The expressions of histone demethylase gene (**A**) and differentially expressed KDM5B (**B**) were obtained from RNA-seq data of mouse peritoneal macrophages treated with LPS and EgCF. Mouse peritoneal macrophages were treated with EgCF (50% v/v) and LPS (1 μg/ml) for 6 h, and mRNA expression levels of KDM5B, KDM6B, and KDM7A (**C**) were detected by qRT-PCR. RAW 264.7 cells were treated with EgCF (50% v/v) and LPS (1 μg/ml) for 6 h, and mRNA levels of KDM5B were measured by qRT-PCR (**D**). Protein levels of KDM5B were measured by Western blotting (**E**). THP-1 cells were treated with EgCF (50% v/v) and LPS (1 μg/ml) for 6 h. mRNA levels of KDM5B were measured by qRT-PCR (**F**), and protein levels of KDM5B were measured by Western blotting (**G**). Protein levels of KDM5B were measured in THP-1 cells stimulated with EgCF (50% v/v) and LPS (1 μg/ml) for 15, 30, 60, 120, and 240 min (**H**). RAW 264.7 cells were treated with cycloheximide (CHX, 50 μg/ml) in combination with EgCF and LPS for 2, 4, and 6 h. Protein levels of KDM5B were measured by Western blotting (**I**). RAW 264.7 cells were treated with the SUMOylation inhibitor TAK-981 (0.5 µM and 1.0 µM) in combination with EgCF and LPS for 6 h, and KDM5B protein levels were measured by Western blotting (**J**). **C**, **D**, **F** Data are presented as the mean + SEM of three independent experiments and compared using the one-way ANOVA and Tukey test. Asterisks indicate a significant difference at *P < 0.05 and **P < 0.01

Histone demethylases could modulate macrophage inflammatory responses by removing methyl groups from lysine and arginine residues [15, 27]. To investigate the relationship between histone demethylases and the anti-inflammatory effects of EgCF, we detected the expression of histone demethylase genes (Fig. 4A) and identified the differentially expressed histone demethylase KDM5B (Fig. 4B) using RNA-seq data of mouse peritoneal macrophages treated with LPS and EgCF. To verify the finding, mouse peritoneal macrophages (PM) stimulated with EgCF and LPS for 6 h were assessed by qRT-PCR. The results showed that the EgCF treatment upregulated the mRNA expression of KDM5B and KDM7A but downregulated the mRNA expression of KDM6B (Fig. 4C). As KDM5B is a demethylase specifically demethylating H3K4me3, we speculated that KDM5B might affect the anti-inflammatory effect of EgCF through regulating H3K4me3 modification. RAW 264.7 cells were also stimulated with EgCF and LPS for 6 h to measure KDM5B mRNA and protein levels by qRT-PCR and Western blotting, respectively. The qRT-PCR results showed that the co-stimulation of LPS and EgCF markedly upregulated the mRNA expression of KDM5B compared with using LPS alone (Fig. 4D), which was similar to the results in mouse peritoneal macrophages (Fig. 4C). Western blotting results showed that the protein level of KDM5B was promoted after EgCF and LPS treatment (Fig. 4E). Similarly, the co-stimulation of EgCF and LPS also markedly upregulated the mRNA and protein expression of KDM5B in THP-1 cells (Fig. 4F and G). Simultaneous stimulation of EgCF and LPS upregulated KDM5B protein expression at different time points in THP-1 cells compared with using the stimulation of LPS alone (Fig. 4H). RAW 264.7 cells were treated with cycloheximide (CHX), an inhibitor of protein synthesis, and we found that KDM5B protein expression was not downregulated when cells were co-stimulated with LPS and EgCF ( Fig. 4I), suggesting that EgCF promoted the stability of KDM5B protein. It has been reported that SUMOylation mediated by the activator STAT 4 protein suppressor (PIAS4) could stabilize KDM5B protein under hypoxia [28]. We then treated RAW 264.7 cells with a SUMOylation inhibitor (TAK-981). The results showed that the protein level of KDM5B was lower in the group of EgCF and LPS co-stimulation after TAK-981 treatment (Fig. 4J), suggesting that SUMOylation was involved in regulating KDM5B. Taken together, these results demonstrated that EgCF promoted KDM5B transcription and protein stability.

### EgCF reduced H3K4me3 enrichment and promoted KDM5B enrichment at the promoters of inflammatory factors

**Fig. 5:**
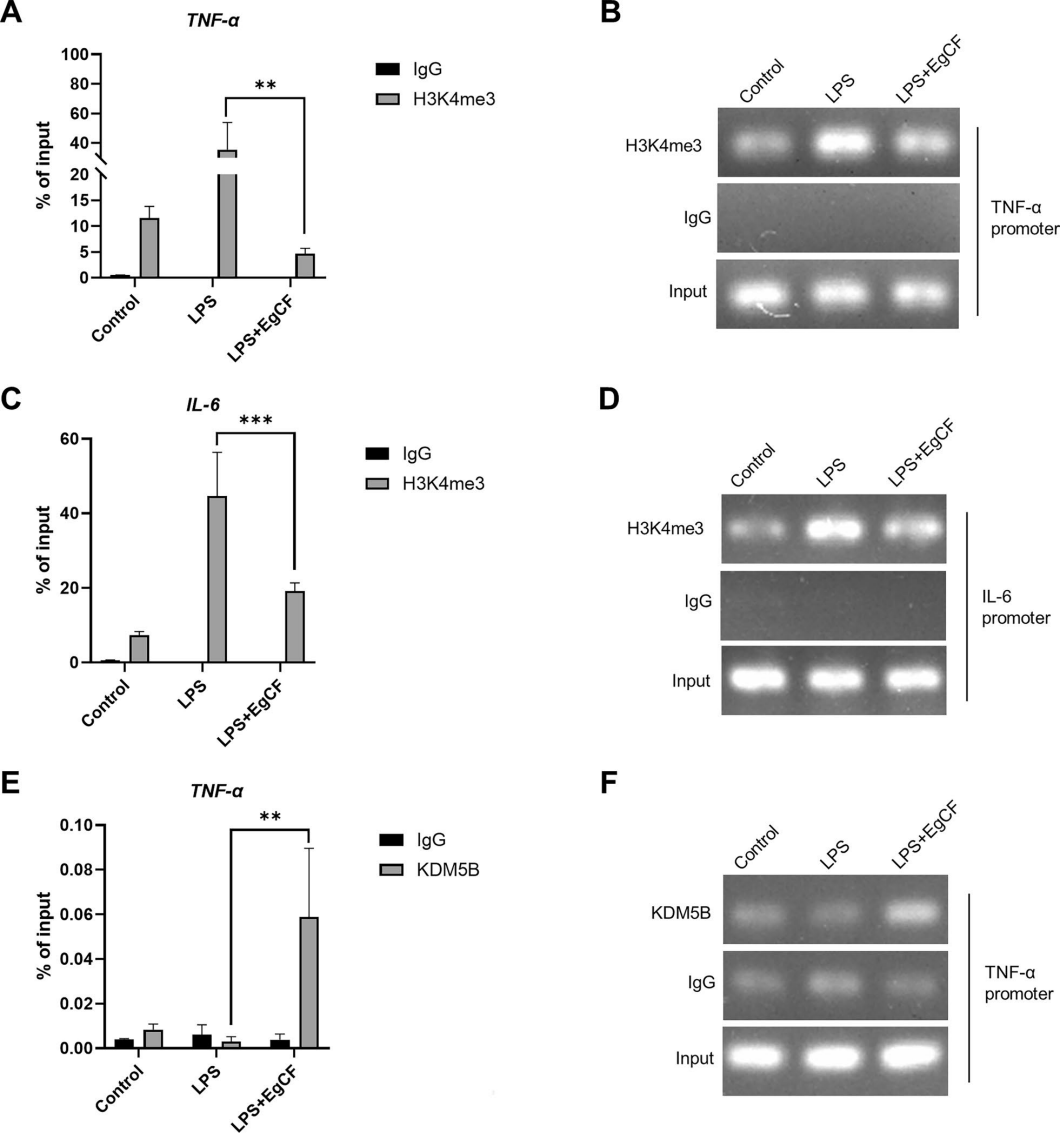
EgCF caused lower H3K4me3 enrichment and higher KDM5B enrichment at the promoters of inflammatory factors. Chromatin immunoprecipitation was performed in RAW 264.7 cells treated with EgCF (50% v/v) and LPS (1 μg/ml) for 6 h. qRT-PCR and semi-qPCR were used to detect the enrichment levels of H3K4me3 and KDM5B at TNF-α and IL-6 promoters (**A**–**F**). Data are presented as the mean + SEM of three replicates, representing one of three independent experiments and compared using the one-way ANOVA and Tukey test. Asterisks indicate a significant difference at **P < 0.01 and ***P < 0.001

To investigate whether KDM5B affected the anti-inflammatory effect of EgCF by regulating H3K4me3 modification, chromatin immunoprecipitation (ChIP) assays were performed in RAW 264.7 cells after stimulation of EgCF and LPS for 6 h. The results showed that compared with using LPS stimulation alone, simultaneous stimulation of EgCF and LPS reduced H3K4me3 enrichment at the TNF-α and IL-6 promoters (Fig. 5A–D). However, simultaneous stimulation of EgCF and LPS increased KDM5B enrichment at the TNF-α promoter in macrophages (Fig. 5E and F). These results demonstrated that EgCF inhibited H3K4me3 enrichment at the inflammatory cytokine promoter via KDM5B in macrophages.

### Inhibition of KDM5B attenuated the anti-inflammatory response of EgCF

**Fig. 6:**
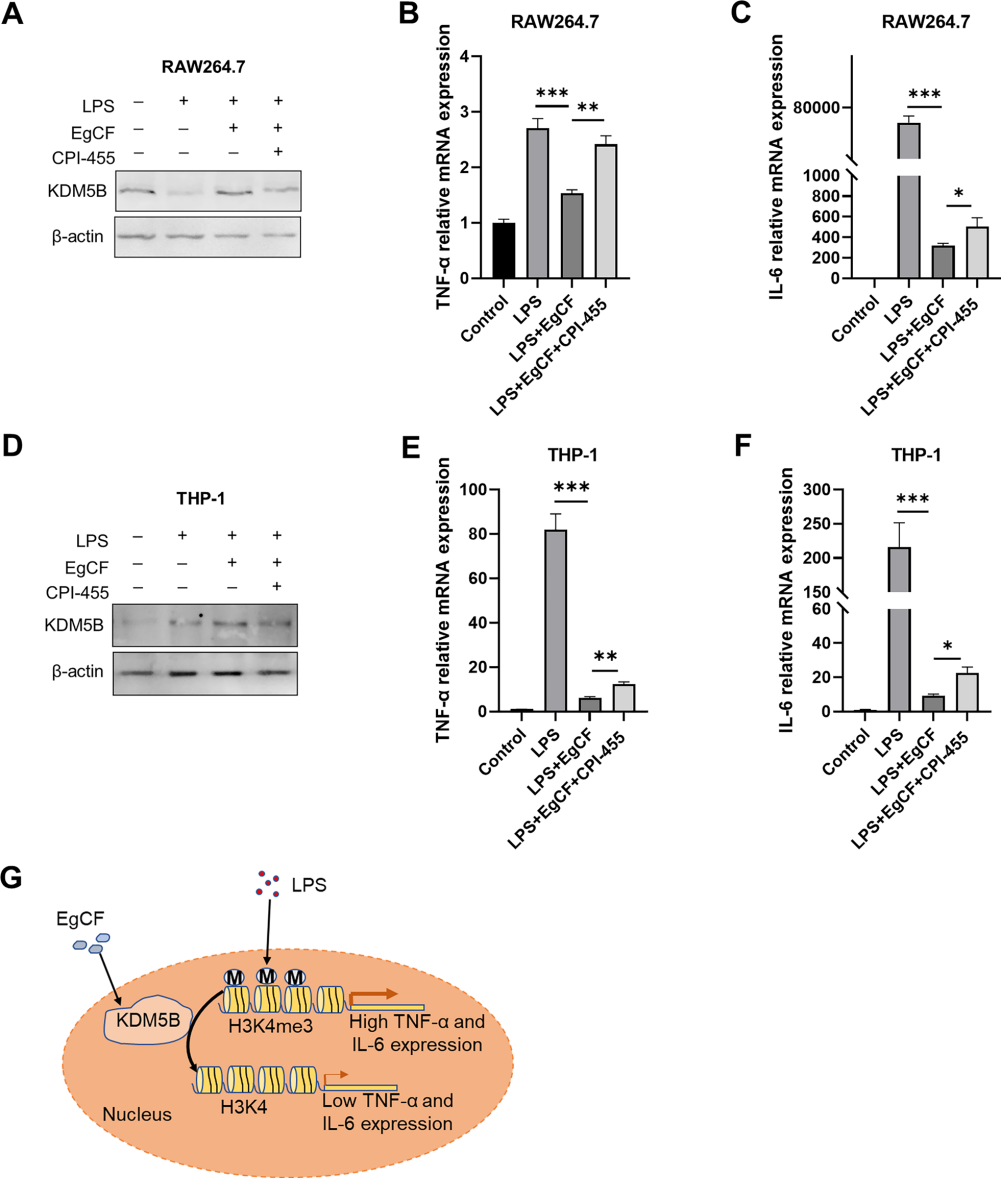
Inhibiting KDM5B attenuated the anti-inflammatory response of EgCF. RAW 264.7 cells were stimulated with EgCF (50% v/v) and LPS (1 μg/ml) for 6 h after pretreatment with CPI-455 (25 μM) for 48 h. Protein levels of KDM5B were measured by Western blotting (**A**). TNF-α and IL-6 mRNA expression levels (**B**, **C**) were measured by qRT-PCR. THP-1 cells pretreated with CPI-455 (25 μM) for 48 h were stimulated with EgCF (50% v/v) and LPS (1 μg/ml) for 6 h. Protein levels of KDM5B were measured by Western blotting (**D**). TNF-α and IL-6 mRNA expression levels (**E**, **F**) were measured by qRT-PCR. Working model for *Echinococcus granulosus* cyst fluid inhibiting the expression of inflammatory factors in macrophages (**G**). **B**, **C, E, F** Data are presented as the mean + SEM of three replicates, representing one of three independent experiments, and compared using the one-way ANOVA and Tukey test. Asterisks indicate a significant difference at *P < 0.05, **P < 0.01, and ***P < 0.001

To verify the mechanism that EgCF promoted KDM5B expression to inhibit the enrichment of H3K4me3 at the promoters of inflammatory cytokines, CPI-455 (a pan-KDM5 inhibitor) was used to pre-treat RAW 264.7 cells for 48 h. RAW 264.7 cells were stimulated with EgCF and LPS for 6 h after CPI-455 treatment (Fig. 6A). The qRT-PCR results demonstrated that the mRNA expression of IL-6 and TNF-α was recovered in RAW 264.7 cells treated with the KDM5 inhibitor CPI-455, indicating that the anti-inflammatory effect of EgCF was attenuated (Fig. 6B and C). CPI-455 was also used to inhibit KDM5B in THP-1 cells (Fig. 6D). Similarly, compared with simultaneous stimulation of EgCF and LPS, the mRNA expression of IL-6 and TNF-α was also significantly upregulated in THP-1 cells treated with CPI-455 (Fig. 6E and F). In summary, inhibiting KDM5B could at least partially abolish the anti-inflammatory effect of EgCF.

## Discussion

The ability of *Echinococcus granulosus* to survive in the host for a long period suggests that the parasite can effectively manage the host's immune response [29]. We have previously shown that EgCF can suppress inflammatory responses by regulating TRAF6 in macrophages [24]. Targeting epigenetic modifiers such as histone demethylases is an effective strategy in drug development. However, the efficacy of histone modification in inhibiting inflammatory response mediated by EgCF in macrophages remains to be explored. In this study, we demonstrated that EgCF promoted the transcription and protein stability of KDM5B, thereby inhibiting the enrichment of H3K4me3 at the promoters of inflammatory factors to suppress inflammatory response (Fig. 6G). Recent evidence also suggests that *Leishmania* parasites mediate epigenetic alterations in macrophages and suppress host defense, thereby facilitating parasite survival and disease progression [30]. *Leishmania donovani* could employ specific histone lysine methyltransferases/demethylases to redirect epigenetic programming of macrophage polarization for its successful establishment within the host [27].

This study used two macrophage models (mouse RAW 264.7 and human THP-1) to investigate the anti-inflammatory effect of EgCF. The results showed that EgCF treatment significantly downregulated the expression of pro-inflammatory cytokines IL-6 and TNF-α in macrophages, similar to the cyst fluid of *E. multilocularis* [31]. Surprisingly, TNF-α in RAW 264.7 cells and IL-6 in THP-1 cells were high in the control group (Fig. 2). Nevertheless, a similar phenomenon has also been observed in other studies [32, 33], and this would not affect the conclusion that the protein levels of TNF-α and IL-6 in cell supernatants were reduced after simultaneous stimulation of EgCF and LPS compared with using LPS stimulation alone.

In general, H3K4me3 is considered a hallmark of active transcription [18]. We found that EgCF suppressed the global levels of H3K4me3 in THP-1 and RAW 264.7 cells induced by LPS, indicating that H3K4me3 was involved in the EgCF regulation of anti-inflammatory responses in macrophages. Based on ChIP-seq data, we also found that H3K4me3 was highly enriched at the pro-inflammatory promoters in human and mouse macrophages, which supported the link between H3K4me3 modification and the anti-inflammatory effect of EgCF.

Subsequently, differentially expressed histone demethylases were analyzed to examine how EgCF downregulated the level of H3K4me3. The results showed that simultaneous stimulation of EgCF and LPS promoted the mRNA and protein expression of KDM5B compared with using LPS stimulation alone [27]. KDM5B is a JmjC-containing demethylase specific to H3K4me3 [34, 35]. In line with our finding, a recent report showed that KDM5B demethylated H3K4me3 at the promoters of IL-12, TNF-α, and arginase-1, thereby suppressing inflammatory responses in a *Leishmania*-infected macrophage model [27]. KDM5B could also inhibit the binding of NF-κB to IL-6 and IL-23a promoters by downregulating H3K4me3 levels in BMDMs [19]. We speculated that EgCF might suppress the H3K4me3 level by promoting the KDM5B level. To investigate whether KDM5B influenced the anti-inflammatory effect of EgCF by regulating H3K4me3 modification, we analyzed the enrichment of H3K4me3 and KDM5B at the promoters of inflammatory factors using ChIP assay. It was found that KDM5B was significantly enriched at the promoters of inflammatory factors after EgCF treatment, while H3K4me3 was the opposite. Previous studies have shown that CPI-455 could mediate pan-KDM5 inhibition and elevate global levels of H3K4me3 [36, 37]. CPI-455 treatment could at least partially abolish the anti-inflammatory effect of EgCF in RAW 264.7 and THP-1 cells. Further development of more potent and selective KDM5B inhibitors might achieve better effects [38].

However, EgCF is a mixture of carbohydrates, lipids, and proteins. For instance, antigen 5 (EgAg5), an abundant highly immunogenic component, is a thermolabile protein [39]; antigen B (EgAgB), one of the major molecules synthesized by the cyst, is a thermostable lipoprotein [40]. Our previous study showed that modification of carbohydrates, but not heat denaturation of proteins, abolished the ability of EgCF to inhibit LPS-induced inflammatory response [12]. Specific components of EgCF that affected the epigenetic control of host macrophages required further exploration. Investigating whether the activity of EgCF is thermostable could provide clues to understanding possible related molecules.

Overall, our findings demonstrated that *E. granulosus* promoted KDM5B expression and suppressed H3K4me3 enrichment at the promoter of inflammatory factors, inhibiting macrophage inflammatory responses. Targeting epigenetic modifiers such as histone demethylases may provide new ideas for the treatment of cystic echinococcosis.

### Supplementary Information

**Additional file 1: Figure S1.** Cell viability after treatment for all cell sources was measured at the indicated time points using the Cell Counting Kit 8 assay (CCK8; Dojindo). ns, not significant.

## Data Availability

Data supporting the conclusions of this article are included within the article.
