# Supplementary figures and images for: Echinococcus granulosus cyst fluid inhibits inflammatory responses through inducing histone demethylase KDM5B in macrophages
Source: Parasit Vectors. 2023 Sep 9;16:321. doi: 10.1186/s13071-023-05948-1 (PMC10492338; doi:10.1186/s13071-023-05948-1)

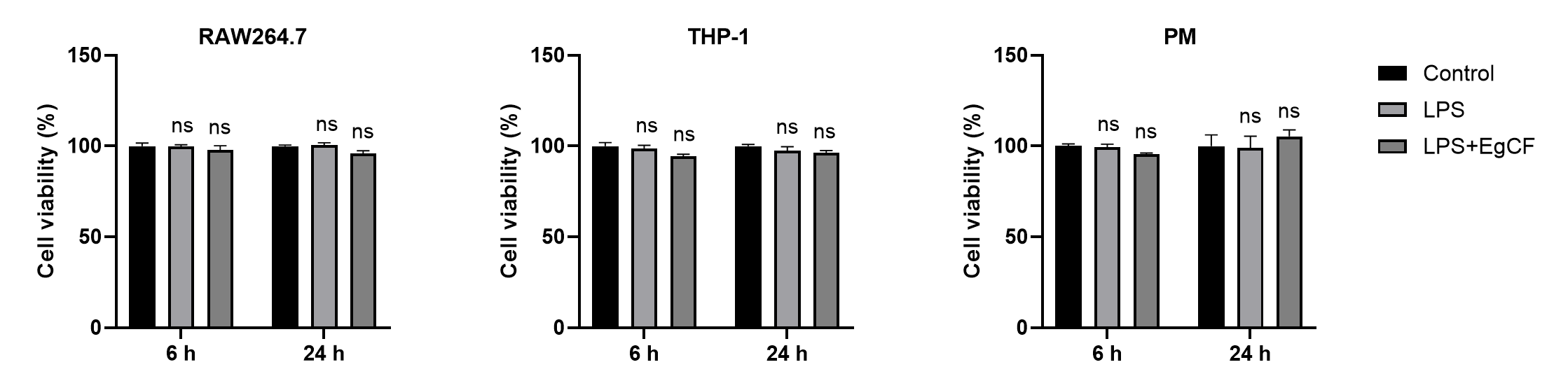

Supplement: Supplementary file 1 — Additional file 1: Figure S1. Cell viability after treatment for all cell sources was measured at the indicated time points using the Cell Counting Kit 8 assay (CCK8; Dojindo). ns, not significant. [file 13071_2023_5948_MOESM1_ESM.tif]
